# Non-thermal plasma induces AKT degradation through turn-on the MUL1 E3 ligase in head and neck cancer

**DOI:** 10.18632/oncotarget.5407

**Published:** 2015-09-30

**Authors:** Sun-Yong Kim, Haeng-Jun Kim, Sung Un Kang, Yang Eun Kim, Ju Kyeong Park, Yoo Seob Shin, Yeon Soo Kim, Keunho Lee, Chul-Ho Kim

**Affiliations:** ^1^ Department of Otolaryngology, Ajou University School of Medicine, Suwon, Republic of Korea; ^2^ Department of Molecular Science and Technology, Ajou University, Suwon, Republic of Korea; ^3^ PSM America Inc., Colorado Springs, CO, USA

**Keywords:** non-thermal plasma (NTP), head and neck cancer (HNC), AKT ubiquitination, liquid type plasma (LTP), mitochondrial E3 ubiquitin protein ligase 1 (MUL1)

## Abstract

Recent research on non-thermal plasma (NTP, an ionized gas) has identified it as a novel cancer therapeutic tool. However, the molecular mechanism remains unclear. In this study, we demonstrated NTP induced cell death of head and neck cancer (HNC) through the AKT ubiquitin–proteasome system. NTP increased the gene expression of mitochondrial E3 ubiquitin protein ligase 1 (MUL1), an E3 ligase for AKT, and NTP-induced HNC cell death was prevented by MUL1 siRNA. We also showed that MUL1 inhibited the level of AKT and p-AKT and MUL1 expression was increased by NTP-induced ROS. Furthermore, we optimized and manufactured a new type of NTP, a liquid type of NTP (LTP). In syngeneic and xenograft *in vivo* tumor models, LTP inhibited tumor progression by increasing the MUL1 level and reducing p-AKT levels, indicating that LTP also has an anti-cancer effect through the same mechanism as that of NTP. Taken together, our results suggest that NTP and LTP have great potential for HNC therapy.

## INTRODUCTION

Non-thermal plasma (NTP) is an ionized gas composed of charged particles, electronically excited atoms and molecules, radicals, and UV photons [1]. The anti-cancer effects of NTP have been reported in several *in vitro* or *in vivo* models and investigated as a novel anti-cancer therapeutic tool [1–4]. Previously, we reported that NTP induced head and neck cancer (HNC) cell apoptosis by DNA damage via the ATM/p53 signaling pathway and cell death through mitogen-activated protein kinase-dependent mitochondrial reactive oxygen species (ROS) [5, 6]. NTP also inhibited thyroid papillary cancer cell invasion via cytoskeletal modulation [7]. However, the molecular mechanism of NTP-induced cancer cell death remains unclear.

AKT is a well-known oncogenic kinase that plays a key role in cell survival, proliferation, apoptosis [8], and tumor development [9]. In relation to HNC, several studies reported the overexpression of AKT and the involvement of kinase activity in the lymph node metastasis of HNC patients [10, 11]. Other reports showed that AKT is involved in the epithelial to mesenchymal transition (EMT) in head and neck squamous cell carcinoma (HNSCC) [12]. Thus, AKT plays a role in the development of HNC, the fifth most common cancer, however, its therapeutic efficiency for HNC through the inhibition of AKT remains unknown.

Herein, for the first time, we investigated the novel anti-cancer mechanisms of NTP through AKT ubiquitination and degradation through MUL1 E3 ligase in HNCs *in vitro* and *in vivo*.

## RESULTS

### NTP induces cell death through K48-linked ubiquitination of active AKT in HNC cells

**Figure 1:**
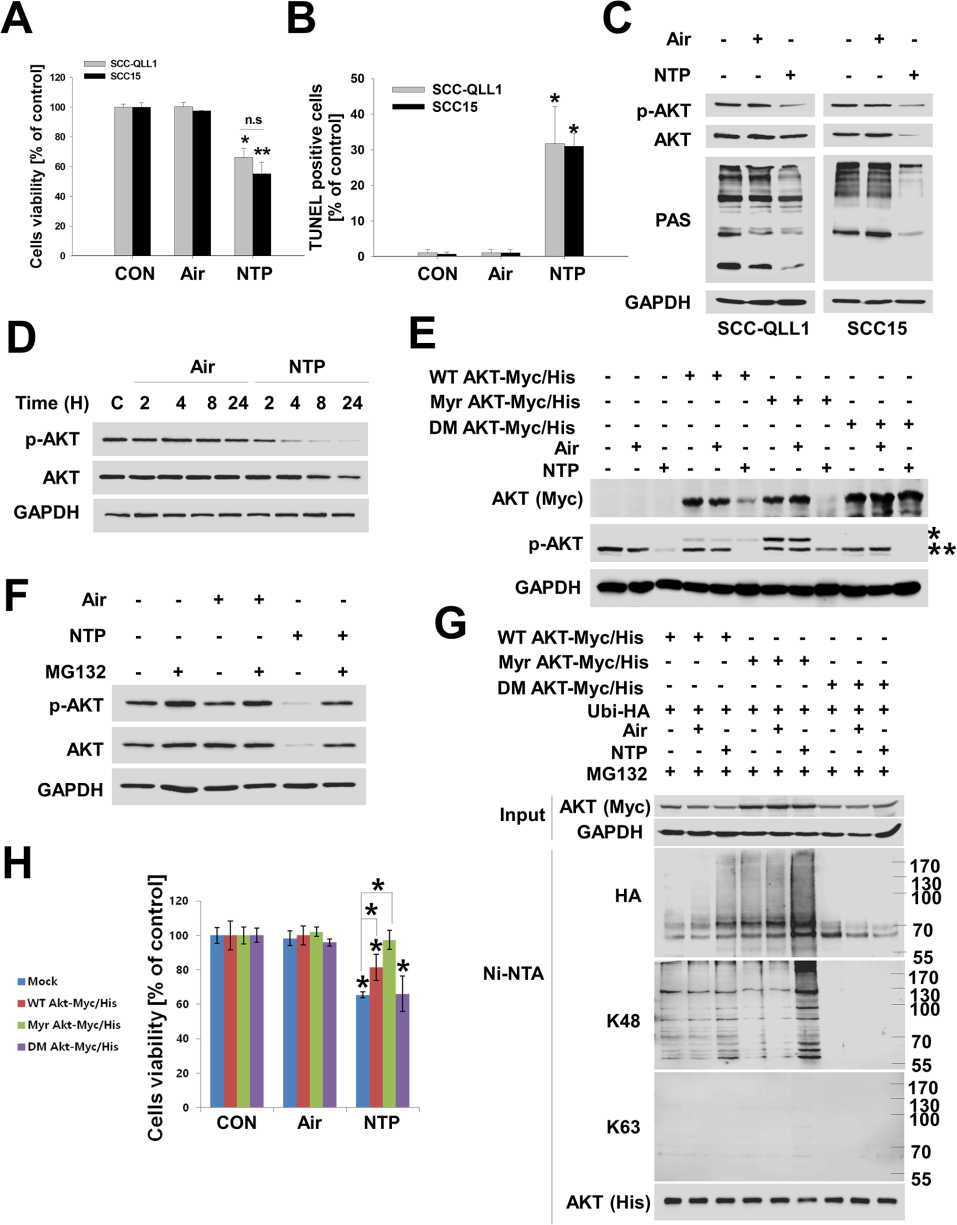
NTP induces cytotoxicity through K48-linked ubiquitination of active AKT in HNC cells **A and B.** NTP-induced cytotoxicity in HNC cells. SCC15 or SCC-QLL1 of human HNC cells was seeded on 48-well plates. Cell viability was evaluated 24 hours after air or NTP treatment using the MTT assay (A, *P* < 0.05, *n* = 6), and apoptotic cell death was analyzed using the TUNEL assay (B, *n* = 3). Asterisks indicate statistically significant differences (*P* < 0.05). Data are expressed as means ± SD. **C.** SCC15 cells were treated with NTP for 24 hours, and AKT, p-AKT, phosphor-AKT substrate (PAS), and GAPDH levels were analyzed by Western blot in the absence of serum. **D.** NTP-induced AKT degradation. To determine time-dependent NTP-induced AKT or p-AKT degradation, SCC15 cells were treated with air or NTP for the indicated times in the absence of serum. Each indicated protein level was evaluated by Western blot. **E.** Active AKT was preferentially degraded by NTP. SCC15 cells were transfected with Myc-His tagged wild-type AKT (WT AKT-Myc/His), constitutively active AKT (Myr AKT-Myc/His), or inactive AKT (T308A/S473A double mutant, DM AKT-Myc/His). A total of 24 hours after transfection, cells were treated with air or NPT, and each indicated protein level was determined by Western blot 24 hours post-air or -NTP treatment. * or ** denotes exo- and endogenous p-AKT, respectively. **F.** Involvement of the proteasome in NTP-induced AKT reduction. SCC15 cells were treated with NTP for 24 hours, and 10 μM MG132 was added 6 hours before cell harvest. **G.** Active AKT was preferentially ubiquitinated by NTP. SCC15 cells were transfected with wild-type AKT (WT AKT-Myc/His), active AKT (Myr AKT-Myc/His), and inactive AKT (DM AKT Myc-His) together with ubiquitin (Ubi-HA) plasmids, after which NTP containing 1 μM MG132 was administered for 24 hours. AKT ubiquitination was determined using Ni-NTA pull-down assays and Western blot. **H.** Active AKT overexpressing cells were protected against NTP-induced cytotoxicity. Each AKT plasmid was transfected into SCC15 cells and treated with NTP. After 24 hours, cell viability was analyzed using the MTT assay (*P* < 0.05, *n* = 6). Asterisks indicate statistically significant differences (*P* < 0.05). Data are expressed as means ± SD.

Followed by our previous studies which demonstrated that NTP induced cell death and apoptosis in several HNC cells [5, 6]. Therefore, we confirmed that NTP was also able to significantly reduce HNC cell viability (Figure 1A), whereas, control gas composed of only helium and oxygen did not reduce cell viability. NTP also caused apoptotic cell death in HNC cells (TUNEL assay, Figure 1B, Supplementary Figure 1A). The AKT kinase plays a role in cell survival, apoptosis, and HNC development [8, 11], thus, we investigated whether NTP-induced cell death correlates positively with the level of AKT. NTP reduced both p-AKT and total AKT (Figure 1C). To explore whether NTP-induced reductions in AKT levels were associated with AKT kinase activity, we examined AKT kinase activity in NTP-treated cells using p-AKT specific-substrate (PAS) antibody. Our results showed not only a reduced amount of p-AKT and total AKT, but also decreased activity of AKT kinase (Figure 1C, PAS blot). In contrast, changes in AKT or p-AKT levels were not observed in gas-treated cells. The reduction in p-AKT and total AKT was induced by NTP in a time-dependent manner (Figure 1D). Interestingly, a reduction in cells of NTP-induced p-AKT was observed after 4 hours of NTP treatment. Thus, we transfected several AKT plasmids including constitutively active AKT (myristoylated AKT, Myr AKT-Myc/His) or double mutant AKT (T308A/S473A double mutant, DM AKT-Myc/His) into SCC15 cells to determine whether NTP preferentially inhibits p-AKT rather than inactive form of AKT. In wild-type AKT (WT AKT-Myc/His)-transfected cells, the overexpressed AKT level (Myc blot) was reduced by NTP treatment. However, a much stronger decrease in AKT levels was observed in the active form of AKT (Myr AKT-Myc/is)-transfected cells treated with NTP. Compared with that of the constitutively active AKT-transfected group, the level of AKT in the inactive form of AKT (DM AKT-Myc/His) was not changed by NTP. Endogenous p-AKT levels were decreased by NTP in Mock and AKT plasmid transfected group (Figure 1E). We then examined the relationship between NTP-induced AKT degradation and the ubiquitin-dependent proteasomal degradation system (UPS) in HNC cells. NTP treatment decreased the level of p-AKT and total AKT, however, NTP-induced AKT reduction was prevented in the presence of MG132, a 26S proteasome inhibitor (Figure 1F). Total- or p-AKT levels were not changed in gas-treated cells. Based on the result that NTP-induced AKT and p-AKT was degraded by UPS, we hypothesized that NTP preferentially ubiquitinated active AKT rather than inactive AKT. To support this hypothesis, Myc and His-tagged wild-type AKT (WT AKT-Myc/His), constitutively active AKT (Myr AKT-Myc/His), or inactive AKT (DM AKT-Myc/His) were transfected into SCC15 cells together with ubiquitin plasmid (Ubi-HA). After 24 hours of transfection, cells were treated with NTP and cultured for 24 hours under serum-free conditions containing MG132. Ubiquitinated AKT was isolated and detected by Ni-NTA His-tag pull-down assays (Figure 1G). In WT AKT-Myc/His transfected cells, ubiquitination of wild-type AKT was induced by NTP treatment. Compared with that of wild-type AKT, the ubiquitination of constitutively active AKT was more highly accumulated in NTP-treated Myr AKT-Myc/His-transfected cells (Figure 1G, lane 3 vs lane 6). AKT ubiquitination was not detected in inactive-form AKT (DM AKT-Myc/His)-transfected cells. AKT could be ubiquitinated via induced lysine 48 (K48)-linked ubiquitination by tetratrico-peptide repeat domain 3 (TTC3) [13] or mitochondrial E3 ubiquitin protein ligase 1 (MUL1) [14] and lysine 63 (K63)-linked ubiquitination by TNF receptor-associated factor 6 (TRAF6) [15]. To determine the type of ubiquitination induced by NTP, we used specific ubiquitin-conjugated protein detection antibodies for K48-linked and K63-linked ubiquintinated proteins (Figure 1G). As NTP-induced AKT ubiquitination patterns showed a co-relationship with K48-linked ubiquitinated protein, K63-linked ubiquitination was not detected in any AKT construct-transfected cells. These results indicated that NTP causes AKT degradation via K48-linked ubiquitination. To support the pivotal role of AKT activity in NTP-induced cell death, we determined the cell viability in several transfected cells with AKT constructs using the MTT assay (Figure 1H). NTP-induced cytotoxicity was increased in the control group (Mock), however, WT AKT-expressing cells were significantly inhibited by NTP-induced cytotoxicity. In addition, Myr AKT-expressing cells were more likely to be rescued from NTP-induced cytotoxicity than were WT AKT-expressing cells in both SCC15 and SCC-QLL1 cells (Supplementary Figure 1B).

### NTP-induced MUL1 expression increases AKT ubiquitination and cytotoxicity

**Figure 2:**
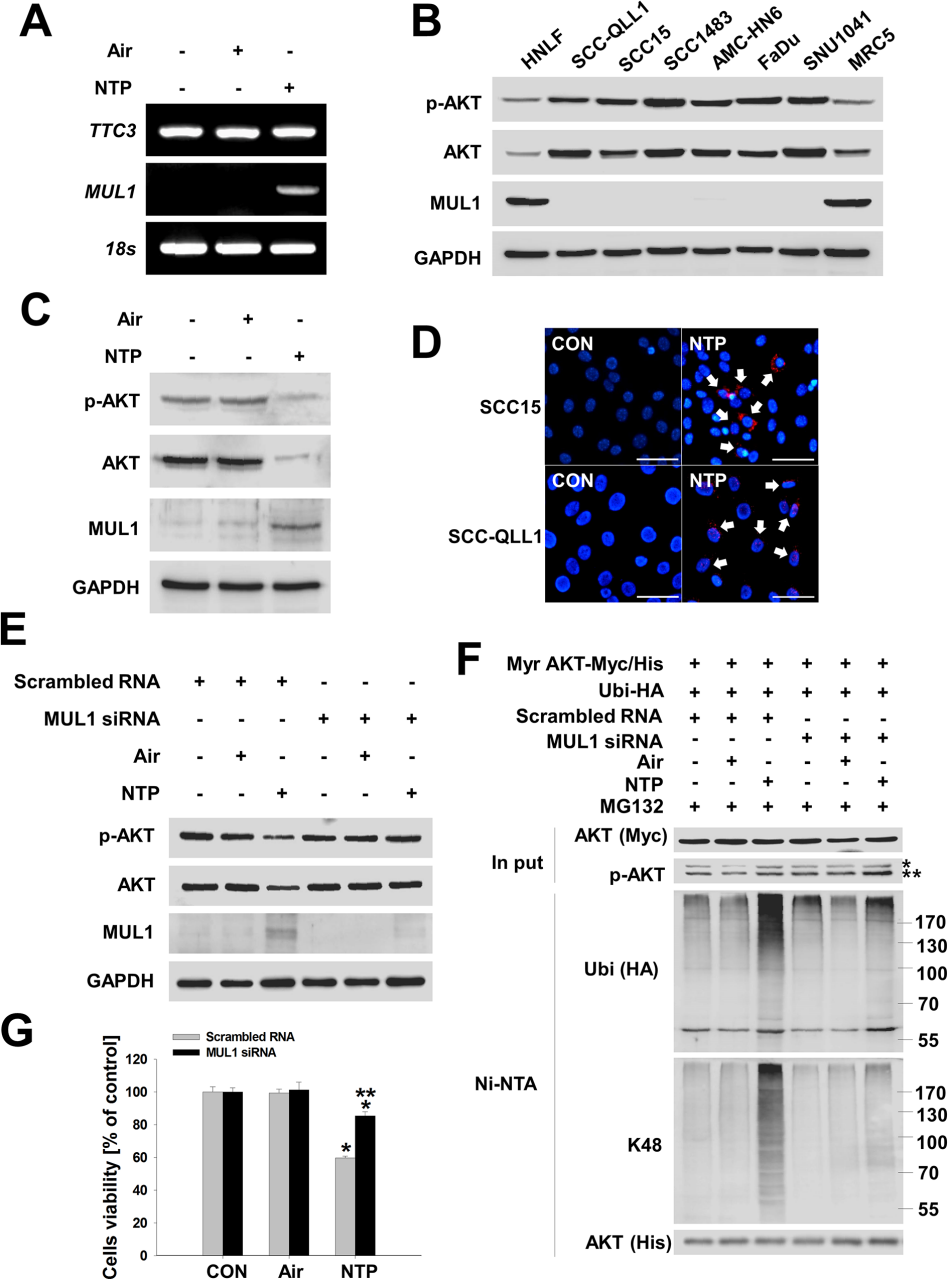
NTP induces MUL1-mediated AKT ubiquitination and degradation **A.** NTP induced increased the level of MUL1 mRNA. SCC15 cells were treated with NTP for 24 hours, after which mRNA levels were evaluated by RT-PCR, respectively. **B.** Determination of MUL1 expression level between normal (HNLF or MRC5) and HNC cell lines. The cell lines information was described in *MATERIALS AND METHODS*. Endogenous AKT, p-AKT, MUL1 and GAPDH was evaluated by Western blot. **C.** SCC15 cells were treated with NTP for 24 hours, after which protein levels were evaluated by Western blot, respectively. **D.** MUL1 bound with AKT by NTP. SCC15 or SCC-QLL1 of HNC cells were attached on glass cover slips, and the cells were treated the next day with NTP in the absence of serum conditions. MUL1-AKT binding was performed using the PLA. Arrows indicate positive signals against the binding between MUL1 and AKT (red). Scale bar represents 50 μm. **E.** NTP-induced AKT reduction was prevented by MUL1 siRNA after transfection with scrambled RNAs or MUL1 siRNA into SCC15 cells and treatment with NTP for 24 hours. Each indicated protein was evaluated by Western blot. **F.** Prevention of NTP-induced MUL1 knockdown. After transfection with scrambled RNAs or MUL1 siRNA, Myr Akt-Myc/His and Ubi-HA or SCC15 cells were treated with NTP for 24 h in the presence of 1 μM MG132. Ubiquitinated Akt was determined by Western blot after Ni-NTA pull-down assays. * or ** denotes exo- and endogenous p-AKT, respectively. **G.** MUL1 knockdown rescued NTP-induced cytotoxicity. Scrambled RNAs or MUL1 siRNA was transfected into SCC15 cells and treated with NTP for 24 hours. Evaluation of cell viability was performed using the MTT assay (*P* < 0.05, *n* = 6). Asterisks indicate statistically significant differences (*P* < 0.05). Data are expressed as means ± SD.

Previous studies have shown that TTC3 or MUL1 induced AKT degradation by UPS via K48-linked ubiqutination [13, 14]. We also observed NTP-induced K48-linked ubiquitination of AKT in HNC cells (Figure 1G). Therefore, to determine the type of E3 ligase against AKT that was involved in NTP-induced AKT ubiquitination, we determined the mRNA levels of TTC3 or MUL1 using RT-PCR in NTP-treated HNC cells (Figure 2A). TTC3 was highly expressed in SCC15 cells, and its expression level was not changed by NTP treatment. In contrast to TTC3 mRNA expression, expression of MUL1 was suppressed in control cells, and NTP induced an increase in MUL1 mRNA levels (Figure 2A). Interestingly, MUL1 expression levels were suppressed in several human HNC cell lines compared with normal cells, such as normal human lung fibroblast (NHLF) or human fetal lung fibroblast (MRC5) (Figure 2B). MUL1 was strongly expressed in only two types of normal fibroblast cells. Consistent with the MUL1 expression pattern, the AKT and p-AKT levels of normal and HNC cells differed. Following MUL1 induction, p-AKT and total AKT levels were decreased by NTP treatment (Figure 2C). To further explore whether NTP-induced AKT degradation was medicated by MUL1, we evaluated the NTP-induced binding between AKT and MUL1 using a proximity ligation assay (PLA) (Figure 2D). In Figure 2A, endogenous MUL1 expression was suppressed in SCC15 cells, but NTP induced an increase in MUL1 expression levels. PLA assays also showed similar protein binding patterns between MUL1 and AKT. We did not observe any PLA-positive fluorescence in control cells, thus, MUL1 and AKT may not bind due to suppressed endogenous MUL1 expression in human HNC cells (Figure 2D, control). However, the PLA-positive signal was observed in NTP-treated cells, possibly increasing the chance for MUL1 and AKT binding by NTP-induced MUL1 expression. NTP-induced AKT degradation was completely prevented by inhibition of NTP-induced K48-linked ubiquitination in MUL1 siRNA-transfected cells (Figure 2E and 2F). Not only was AKT degradation inhibited via K48-linked ubiquitination, but cell viability was rescued from NTP-induced cytotoxicity by MUL1 siRNA (Figure 2G). These results suggest that NTP-induced MUL1 expression may play a pivotal role in HNC cell death through AKT degradation and that MUL1 could contribute to HNC development via sustained AKT activity.

### NTP-induced cellular ROS increase MUL1/AKT binding and cytotoxicity

**Figure 3:**
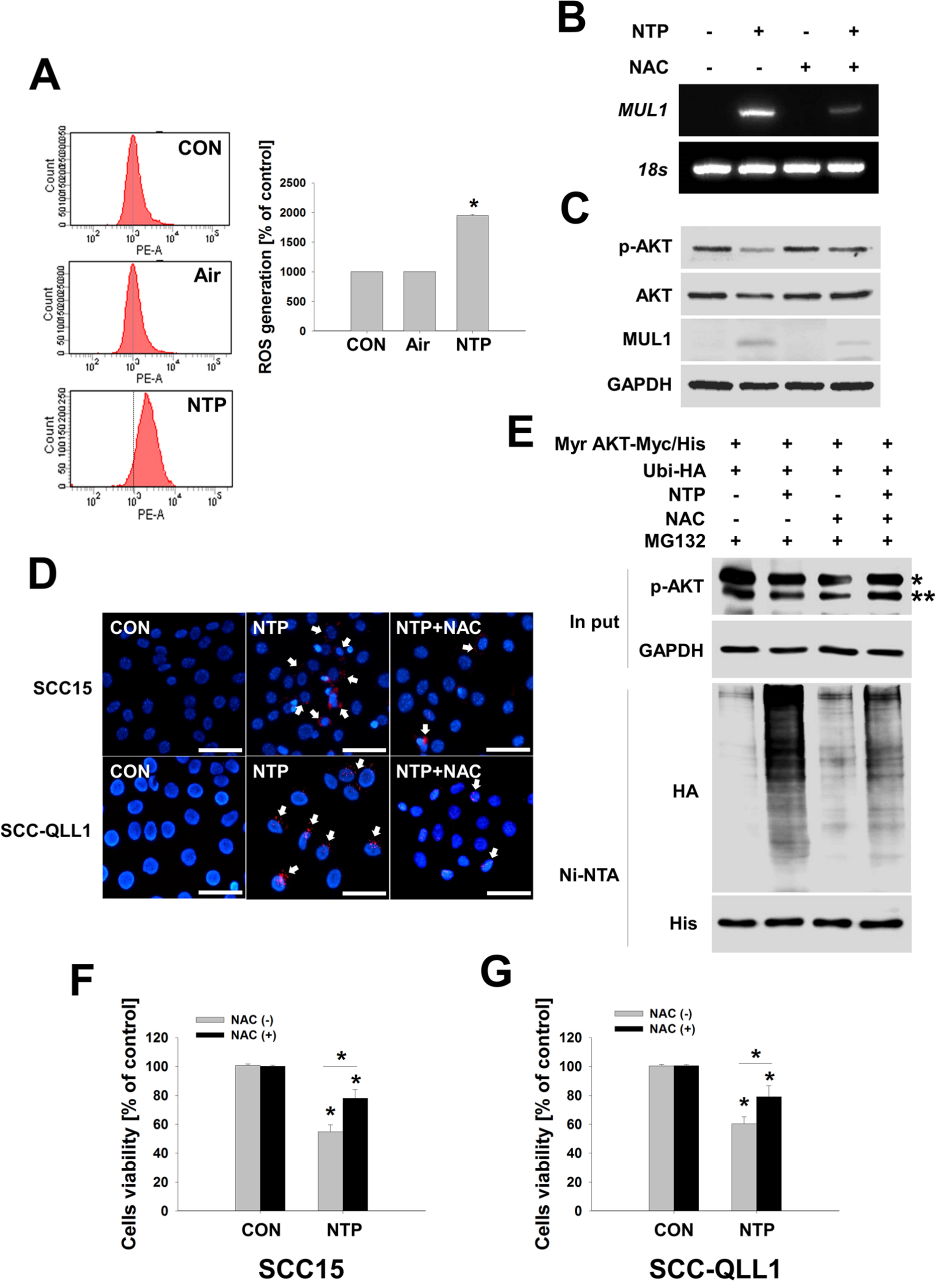
ROS plays a role in NTP-induced AKT stability and MUL1 expression **A.** Measurements of cellular ROS. SCC15 cells were treated with NTP for 24 hours, and cellular ROS was measured using FACS analysis (* vs CON, *P* < 0.05, *n* = 3). Asterisks indicate statistically significant differences (*P* < 0.05). Data are expressed as means ± SD. **B and C.** NAC inhibited MUL1 gene expression and AKT degradation by NTP. NTP treated for 24 hours at SCC15 with or without NAC. NAC (10 mM) was pretreated for 1 hour before NTP treatment. Each indicated mRNA and protein was evaluated by RT-PCR and Western blot. **D.** NTP-induced binding between MUL1 and AKT was inhibited by NAC. SCC15 cells were seeded on cover glass, after which NTP was treated for 24 hours. MUL1 and AKT binding was examined using the PLA assay. Arrows indicate positive signals against the binding between MUL1 and AKT (Red, scale bar = 50 μm). **E.** SCC15 cells were transfected with Myr AKT-Myc/His and Ubi-HA plasmids. After 24 hours, cells were pretreated with 10 mM NAC, and NTP was treated for another 24 hours in the absence of serum containing 1 μM MG132. Ubiquitinated AKT was identified by Ni-NTA His pull-down assay and Western blot. * or ** denotes exo- and endogenous p-AKT, respectively. **F and G.** SCC15 or SCC-QLL1 cells were seeded on 48-well plates, after which 10 mM of NAC was pretreated for 30 min before NTP treatment. NTP was treated for 24 hours in the absence of serum, and cells viability was evaluated using the MTT assay. Asterisks indicate statistically significant differences (*P* < 0.05, *n* = 6). Data are expressed as means ± SD.

Our previous reports showed that NTP induced increases in cellular ROS, contributing to the cell death of HNC [6]. In this study, we also explored whether NTP induced ROS production in human HNC cells. In Figure 3A, cellular ROS was increased in NTP-treated SCC15. NTP-induced ROS increased MUL1 gene expression and N-acetyl cysteine (NAC), a general anti-oxidant chemical, and inhibited NTP-induced MUL1 induction (Figure 3B). NTP-induced cellular ROS was also involved in MUL1-mediated AKT degradation and these event was prevented by NAC pretreatment (Figure 3C). NAC also inhibited the NTP- induced binding between AKT and MUL1 as well as AKT ubiquitination (Figure 3D and 3E). Both SCC15 and SCC-QLL1 human HNC cells were rescued from NTP-induced cytotoxicity by pretreatment with NAC (Figure 3F and 3G). These results indicated that NTP-induced cellular ROS plays a pivotal role in HNC cell death through MUL1 induction and AKT ubiquitination.

### A liquid type of NTP (LTP) maintains anti-cancer activity

**Figure 4:**
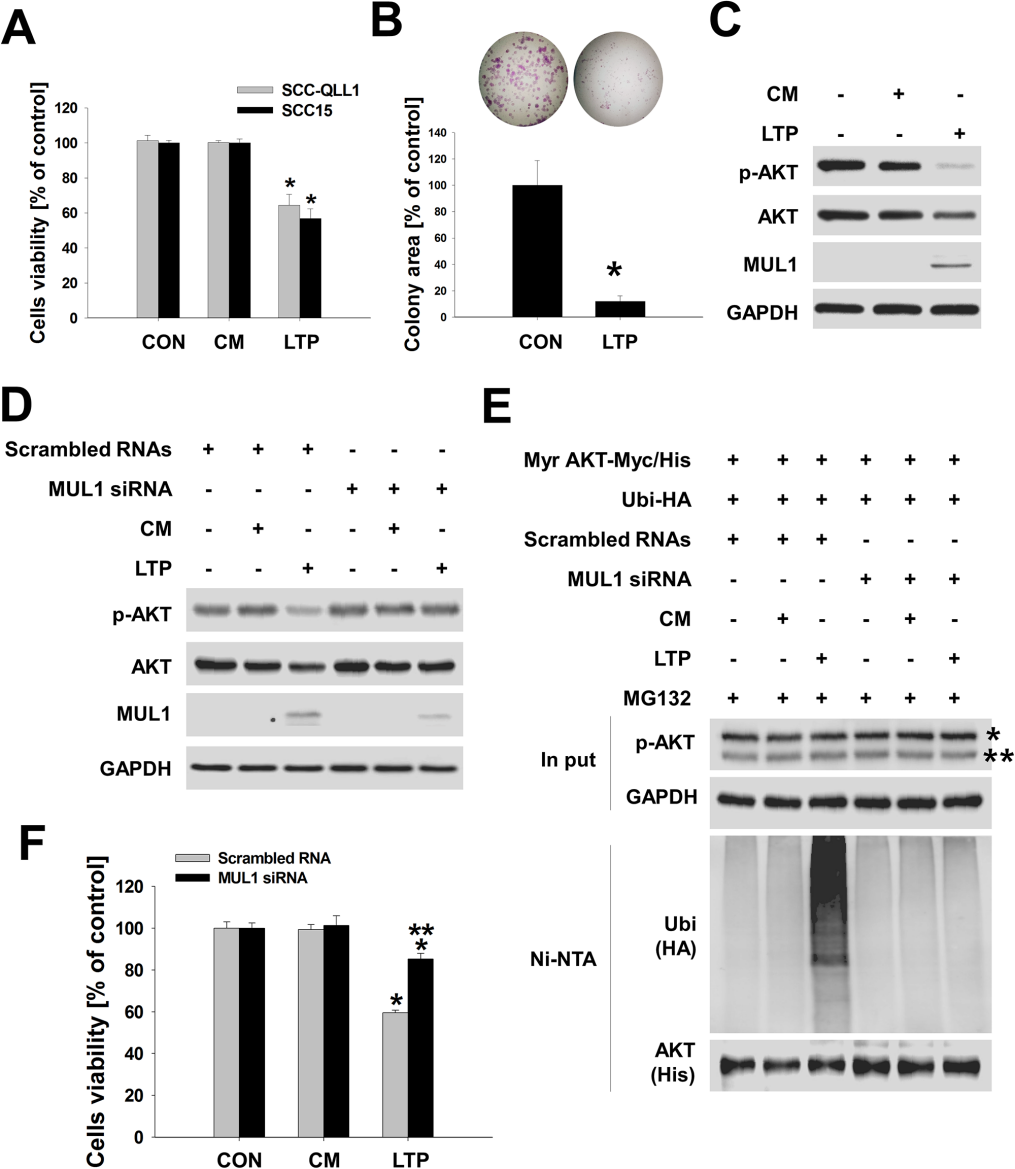
LTP has anti-cancer effects **A.** SCC15 cells seeded on 48-well plates and LTP treated for 24 hours in the absence of serum. After 24 hours, cell viability was examined using the MTT assay (*n* = 6). Asterisks indicate statistically significant differences (*P* < 0.05). Data are expressed as means ± SD. **B.** Total of 1,000 cells of SCC15 seeded on six-well plates and LTP containing 10% serum were replaced every other day. Colony forming was stained with crystal violet and quantified (*n* = 3). Asterisks indicate statistically significant differences (A and B, *P* < 0.05) between LTP- untreated and treated groups. Data are expressed as means ± SD. **C.** LTP was administered to SCC15 cells, and each indicated protein was determined using Western blot. **D.** LTP-induced MUL1-mediated AKT degradation. LTP was treated with scrambled RNA or MUL1 siRNA transfected cells for 24 hours. Air-treated media (control media, CM) was prepared in the absence of NTP. Each indicated protein level was determined using Western blot. **E.** MUL1-mediated AKT ubiquitination was induced by LTP. Scrambled RNA or MUL1 siRNA was transfected into SCC15 cells, after which active AKT (Myr AKT-Myc/His) was transfected together with ubiquitin (Ubi-HA). LTP was treated to transfected cells for an additional 24 hours in the presence of 1 μM MG132, and AKT ubiquitination was evaluated based on Ni-NTA pull-down assays. **F.** LTP-induced cytotoxicity was prevented by MUL1 siRNA. Scrambled RNA or MUL1 transfected into SCC15 cells were seeded on 48-well plates and cell viability was determined using the MTT assay (*n* = 6). Asterisks indicate statistically significant differences (*P* < 0.05) between LTP -untreated and LTP treated groups (* vs CON; ** vs scrambled RNA). Data are expressed as means ± SD.

Our previous studies and this study showed that NTP killed HNC cells *in vitro* and *in vivo* using the direct treatment method [5, 6]. However, we considered developing a novel type of NTP that would allow easy delivery *in vivo* while offering similar or more potent anti-cancer effects. Thus, we manufactured a liquid type of NTP (LTP) and validated NTP anti-cancer effects through MUL1-mediated AKT ubiquitination in HNC cells. We prepared LTP under several different conditions, changing the distance or time of NTP treatment. For optimization of LTP, we measured the concentration of ozone, ultraviolet A (UV-A), and ultraviolet B (UV-B) in each LTP under different conditions (Supplementary Figure 2, Supplementary Table 1). Finally, we manufactured LTP such as culture media (15 ml) were treated with NTP for 15 minutes at a distance of 1~2 cm from the media (Supplementary Figure 3A). Air-treated media were prepared in the same way for LTP preparation in the absence of NTP. We used air-treated media (control media, CM) as a negative control against LTP. We measured ROS such as oxygen, hydrogen, ozone, or nitrate in LTP (Supplementary Table 2). The pH of air-only or LTP did not change during LTP preparation. Manufactured LTP decreased cells viability in human HNC cell lines (Figure 4A). The colony-forming growth of HNC cells was strongly inhibited in LTP-treated groups compared with the CM (Figure 4B, Supplementary Figure 3B). LTP also reduced AKT or p-AKT levels and increased the level of MUL1 (Figure 4C). CM did not change the levels of AKT, p-AKT, or MUL1. In addition, LTP induced a reduction in AKT and p-AKT levels, which was prevented by MUL1 knockdown (Figure 4D). To confirm MUL1-mediated AKT ubiquitination, SCC15 cells were transfected with MUL1 siRNA, active AKT (Myr AKT-Myc/His), and ubiquitin (Ubi-HA) plasmids (Figure 4E). Whereas LTP strongly induced AKT ubiquitination in SCC15 transfected with scrambled RNAs compared with CM-treated SCC15 (Figure 4E, second vs. third lane), LTP-induced AKT ubiquitination was significantly suppressed in SCC15 transfected with MUL1 siRNA (Figure 4E, third vs. sixth lane). In addition to inhibiting AKT ubiquitination, MUL1 knockdown prevented LTP-induced cytotoxicity and was not able to not suppress colony-forming growth by LTP compared with scrambled RNA-transfected cells (Figure 4F, Supplementary Figure 3C). These results indicated that LTP maintains its anti-cancer effect through MUL1-mediated AKT ubiquitination.

### LPT strongly inhibits the development of HNC *in vivo*

**Figure 5:**
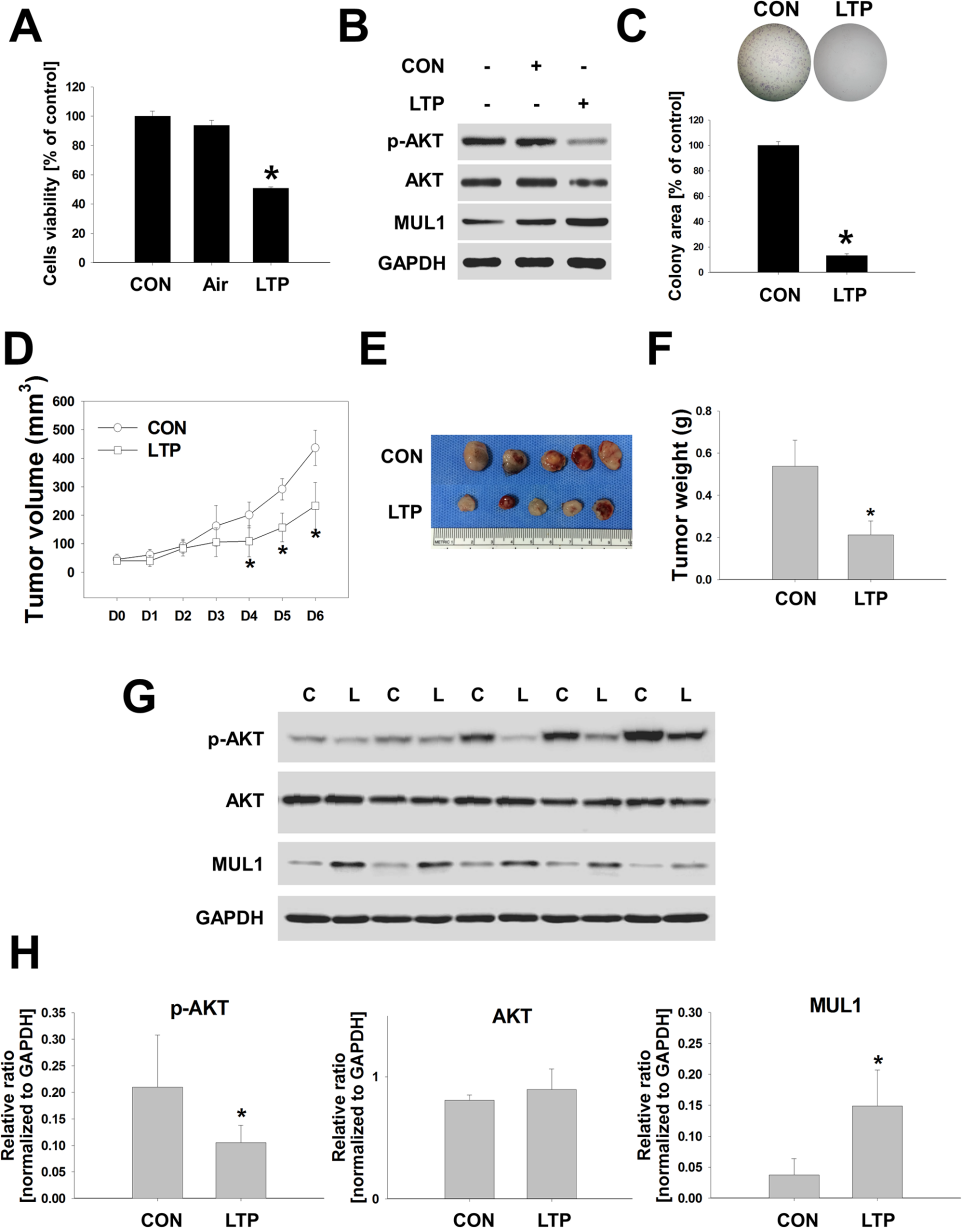
LTP inhibites tumor growth in a syngeneic mouse model SCC7, murine HNC cells, were treated with LTP for 24 hours in the absence of serum. **A.** Cell viability was evaluated using the MTT assay (*n* = 6). Asterisks indicate statistically significant differences (*P* < 0.05). Data are expressed as means ± SD. **B.** p-AKT, AKT, MUL1, and GAPDH were analyzed by Western blot. **C.** Colony-forming growth was determined and quantified by crystal violet staining (*n* = 3). Asterisks indicate statistically significant differences (*P* < 0.05). Data are expressed as means ± SD. **D to H.** Murine HNC cells of SCC7 (1 × 10^6^ cells) were inoculated subcutaneously in a C3H/HeJ mouse and treated with LTP for 6 days. Tumor volume (D), final tumor images (E) and weight (F). (G and H), p-AKT, AKT, MUL1, and GAPDH was quantified by Western blot. Asterisks indicate statistically significant differences (*P* < 0.05, *n* = 5) between LTP-untreated and treated groups. Data are expressed as means ± SD.

In this study, we showed that NTP and LTP, a liquid type of NTP, have anti-cancer effect through MUL1-induced AKT degradation *in vitro*. Therefore, we confirmed the anti-cancer effect of LTP *in vivo* using two types of mouse tumor models. To test LTP anti-cancer effect in a syngeneic mouse tumor model, we explored whether LTP influenced the biological effects of murine HNCs, such as cell viability, AKT degradation, or colony-forming growth. LTP reduced the murine HNC cells of SCC7 as well as AKT or p-AKT levels (Figure 5A and 5B). Murine MUL1 was increased by LTP, however, endogenous MUL1 was also detected. The colony-forming growth of SCC7 was strongly inhibited by LTP (Figure 5C). Based on these results, we subcutaneously administrated SCC7 cells into C3H/HeJ mice and treated them with LTP for a week. LTP showed an inhibitory effect against tumor development after the fourth treatment (Figure 5D). After the final treatment of LTP, tumor weight was significantly reduced in the LTP-treated group compared with the control-media-injection group (Figure 5E and 5F). Based on biochemical analysis data, LTP induced a reduction in p-AKT or increased the level of MUL1 (Figure 5G and 5H), but the total AKT levels were not changed.

**Figure 6:**
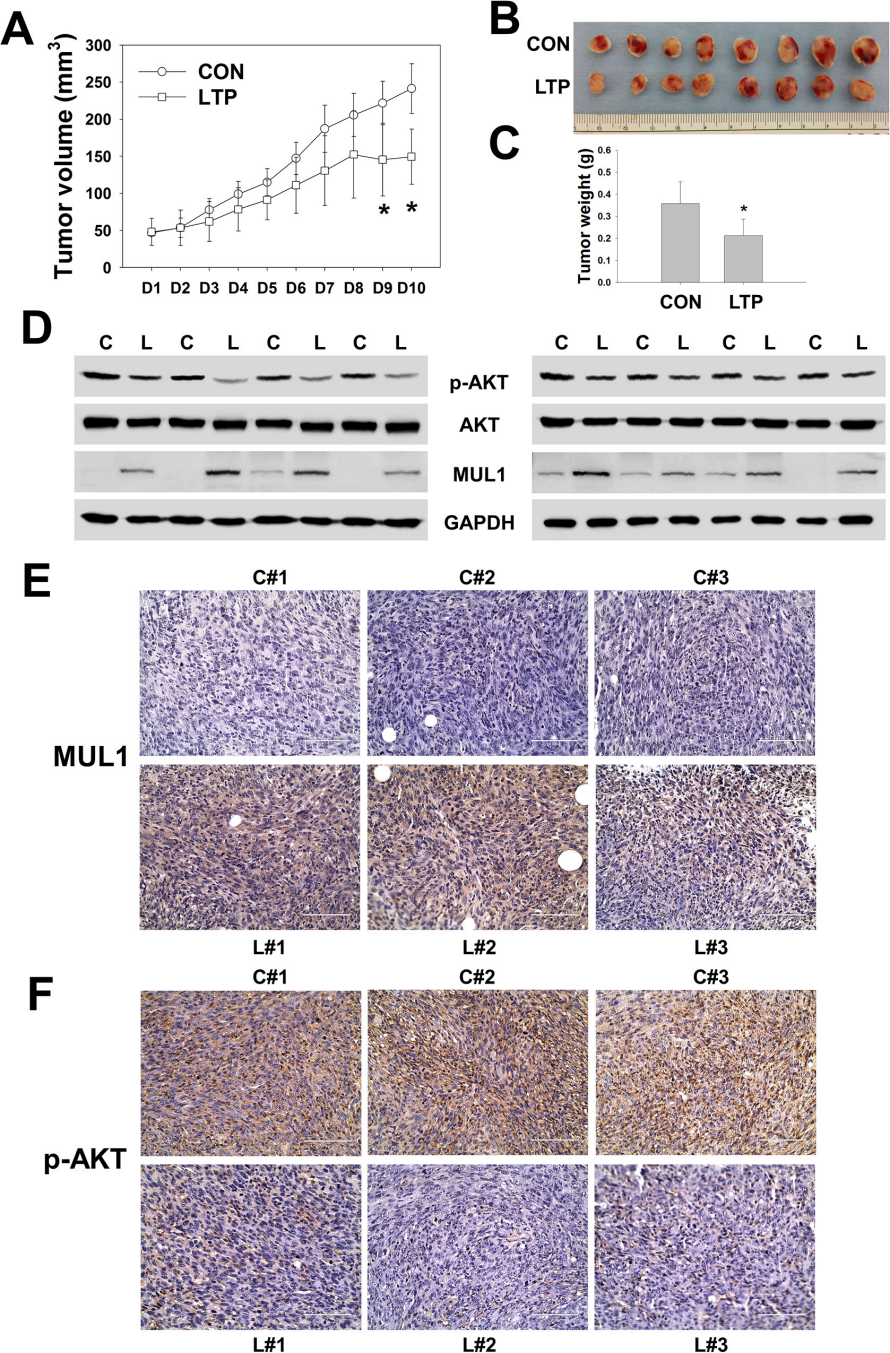
LTP shows anti-cancer effect in xenograft *in vivo* model SCC15 (2 × 10^6^ cells) of human HNC cells were administrated subcutaneously to BALB/c nu/nu mice and treated with LTP every day for 10 days. **A.** Tumor volume. **B and C.** Final tumor images and weight. Asterisks indicate statistically significant differences (*P* < 0.05, *n* = 8) between LTP -untreated and treated groups (A and C). Data are expressed as means ± SD. **D.** p-AKT, AKT, MUL1, and GAPDH was performed by Western blot. Each proteins level was quantified and represented in Supplementary Figure 4. **E. and F.** Immunohistochemistry analysis of MUL1 (E) or p-AKT (F).

To confirm the LTP anti-cancer effects in a syngeneic tumor model, we subcutaneously inoculated human HNC cells of SCC15 into BALB/c nu/nu mouse with LTP treatment in the same manner. In xenograft models, LTP was administered 10 times due to the gradual formation of SCC15 cells with tumors compared with the syngeneic model. Significant inhibition in tumor volume was detected after the ninth treatment of LTP (Figure 6A). In the LTP-treated group, a significant reduction in p-AKT levels was observed, whereas MUL 1 levels were increased by LTP compared with the control (Figure 6D, Supplementary Figure 4A and 4C). Similar to syngeneic *in vivo* results, total AKT levels were not changed by LTP in xenograft models (Figure 6D, Supplementary Figure 4B). Based on immunohistochemistry analysis, MUL1 expression was increased in LTP-treated tumors (Figure 6E). p-AKT levels were strongly stained in the control groups; however, LTP induced a reduction in p-AKT-positive staining (Figure 6F). These results suggested that NTP or LTP possesses anti-cancer effects and could be considered a novel therapeutic tool for HNC therapy.

## DISCUSSION

Surgical treatment for HNC can be a formidable task, especially when the cancer occurs near sites correlated to breathing, vocalization, and swallowing. Postoperatively, patients may suffer from various anatomical and functional disabilities. Other treatment strategies such as chemotherapy (5-FU, cisplatin, methotrexate, gemcitabine, cytarabine, paclitaxel, and vinorelbine), monoclonal antibody (Cetuximab, anti-EGFR monoclonal antibody), and radiotherapy are now widely recognized as part of the standard treatment for HNC. However, recent studies have reported on the incidence of patients showing resistance to chemo-, antibody-, and radiotherapy [16]. For these reasons, the 5-year survival rate has not improved significantly in many advanced HNC. Therefore, the development of a novel therapeutic tool that can enhance treatment efficiency and preserve organ function in HNC is urgently required.

Today, many research groups are active in plasma medicine as NTP application in biology and medicine also belongs in this field. NTP considered as the fourth state of the matter, already is used widely in industrial and medical applications. Recently, the development of revolutionary new plasma devices generating NTP has extended its potential applications especially in biology and medicine [17–19]. Some studies have shown that NTP has an anti-cancer effect against solid tumors such as skin, liver, and colon cancer [2–4]. However, in this study, we developed a new type of NTP such as LTP for easy delivery to insight of cancer and determined the anti-cancer effect of LTP *in vitro* and *in vivo*. One of the most remarkable characteristics of the use of NTP or LTP for cancer therapy is its ability to kill cancer cells selectively. NTP caused a few cell deaths in normal cell lines such as HNLF. However, no significant differences were observed with regard to LTP (Supplementary Figure 5). In addition, LTP did not show any signs of embryotoxicity based on the fish embryo toxicity test (FET) using the zebrafish model (Supplementary Figure 6). These results indicate that LTP or NTP could be used as novel therapeutic tools without causing normal tissue damage.

The serine/threonine kinase AKT, also known as PKB (protein kinase B), regulates and plays a role in cell survival, death, cancer development [8, 9]. However, little is known regarding AKT and its inhibitory effect in physiology correlated to HNC therapy. Recent studies have reported that BYL719, a specific inhibitor of phosphatidylinositol 3-kinase (PI3K) p110α, showed anti-cancer effects in HNSCC cells *in vitro* [20]. PI3K is a well-known protein upstream of AKT. A phase I study of BYL719 using cetuximab and radiation therapy in patients with stage III/IVB HNC is in progress.

According to a recent study, MUL1 acts as a novel E3 ligase and negatively regulates AKT signaling [14]. The study demonstrated that MUL1 bound with the kinase domain of fully phosphorylated AKT and inhibited AKT signaling, leading to suppression of cell proliferation and migration through K48-linked ubiquitination of AKT. It is also known that MUL1 promotes stress-induced mitochondrial hyperfusion (SIMH) accompanied by NF-kB activation under specific physiological conditions [21]. Mitophagy was also increased by deregulation of MUL1 through inactivation of Omi/HtrA2 protease, which has pro-survival functions, and overexpression of MUL1 in skeletal muscle and myoblast cultures was sufficient for the induction of mitophagy [22, 23]. Previously, we reported that NTP resulted in alterations in the mitochondrial membrane potential (MMP) and accumulation of intracellular ROS generated from the mitochondria in HNC cells [6]. The study also showed that NTP induced increased level of cellular ROS and MUL1 expression, which resulted in decrease levels of p-AKT and AKT. In addition, the blocking ROS production by NAC inhibited NTP- or LTP-induced cell death through AKT degradation by MUL1, implying that ROS is at least one of the major factors for NTP- or LTP-induced cell death.

In this study, we showed that NTP and LTP induced AKT degradation by MUL1, which suggests that MUL1 plays a pivotal role in HNC cell death. Previous studies have shown that Growth Inhibition and Death E3 Ligase (GIDE), another name for MUL1, induced cell apoptosis and slowed growth [24]. The authors suggested that these pro-apoptotic or growth inhibition effects of GIDE may account for its absence in tumor cells. In addition, another recent study demonstrated that MUL1 DNA methylation is a potential biomarker in Iranian females with cervical intraepithelial neoplasia and dysplasia [25]. In the case of HNC, the most common risk factors, such as cigarette smoking or alcohol use, may induce MUL1 DNA methylation, ultimately leading to MUL1 suppression in the human HNC cell lines (Figure 2B).

Herein, we provided the first demonstration that NTP- or LTP-induced AKT degradation is mediated by MUL1. Based on these results, we suggest that NTP or LTP could be used as a novel therapeutic tool for HNC therapy, acting through AKT ubiquitination and degradation mediated by MUL1 expression turn-on.

## MATERIALS AND METHODS

### Cells

Head and neck cell lines originating from human oral cavity cancer (SCC15) and human hypopharynx cancer cells (FaDu) were purchased from the American Type Culture Collection (ATCC, Manassas, VA, USA). Human oral cavity cancer (SCC-QLL1 and SCC1483) and SNU1041 human hypopharynx cancer cells, SCC7 murine squamous carcinoma cells and human lung fibroblast (MRC-5) were from the Korean Cell Line Bank (KCLB, Seoul, Korea). AMC-HN6 (floor of mouth cancer) was kindly provided by Dr. Sang-Yoon Kim (Asan Medical Center, University of Ulsan College of Medicine). Human normal lung fibroblast (HNLF) was purchased from Lonza (Walkersville, MD, USA). SCC-QLL1, FaDu, HN6, and MRC5 cells were grown in Minimum Essential Medium (MEM; GIBCO, Carlsbad, CA, USA) supplemented with 10% fetal bovine serum (FBS) and penicillin–streptomycin at 100 μ/mL (GIBCO). SCC15 and SCC1483 cells were maintained in Dulbecco's Modified Eagle's Medium: Nutrient Mixture F-12 (DMEM/F12; GIBCO, Carlsbad, CA, USA) supplemented with 10% fetal bovine serum (FBS) and penicillin–streptomycin at 100 μ/mL (GIBCO). SCC7 cells were maintained in Roswell Park Memorial Institute (*RPMI*) *1640* (GIBCO, Carlsbad, CA, USA) supplemented with 10% fetal bovine serum (FBS) and penicillin–streptomycin at 100 μ/mL (GIBCO) at 37°C with 5% CO_2_ under humidified conditions.

### Plasmids, reagents, and antibodies

Wild-type AKT (WT AKT-Myc/His), constitutively active AKT (myristoylated AKT, Myr AKT-Myc/His), double mutant AKT (T308A/S473A double mutant, DM AKT-Myc/His), and ubiquitin-hemagglutinin (Ubi-HA) plasmids were kindly provided by Prof. Yun-Song Lee (Samsung Biomedical Research Institute, Sungkyunkwan University School of Medicine) [26]. MG132 and N-acetyl cysteine (NAC) were purchased from Sigma (St. Louis, MO, USA). Antibodies were obtained from the following sources: anti-AKT, anti-p-AKT (Ser473, for Western blot), pan anti-p-AKT (Ser473, for immunohistochemistry), anti-Phospho-AKT Substrate (PAS), anti-Myc-tag, anti-His-tag, anti-HA-tag, HRP-conjugated anti-mouse IgG, anti-rabbit IgG (Cell Signaling, Beverly, MA, USA), anti-MUL1 (Abcam, Cambridge, MA, USA), anti-lysin-48-linked ubiquitin, and anti-lysin-63-linked ubiquitin (Millipore Corporation, Bedford, MA, USA).

### Experimental system specifications of NTP

The plasma device was designed and manufactured as a spray-type atmospheric pressure NTP system with a newly designed arc-free and antistatic plate to provide uniform NTP for biological research applications. The plasma source was equipped with a pair of electrodes made of Al_2_O_3_ (high-voltage and ground electrodes, 10 × 40 mm^2^ in dimension, 2-mm gap between electrodes) isolated from direct contact with the plasma using a ceramic barrier. The specifications of the power supply with this system are 2 kV minimum, 13 kV maximum, and mean frequency 20–30 kHz; these specifications can vary with the type and amount of gas used. In this study, helium (He) and oxygen (O_2_) were used as carrier gases because we previously showed that the addition of O_2_ to He plasma improved the efficiency of cancer cell inhibition. The voltage and current of NTP were measured uniformly and stably. The plasma density using He + O_2_ as a carrier gas was calculated as ~10^6^/m^3^ based on optical emission spectroscopy, and the ROS density was ~10^13^/m^3^. The temperature of plasma gas was kept low, at ~35°C, even after 10 min at 13 kV for NTP treatment. The distance between the plasma device and the bottom of the culture dish was maintained at around 2 cm. The NTP jet partially dispelled the media.

### Liquid-type NTP (LTP) preparation

The method used to prepare liquid-type NTP (LTP) was optimized by testing under several different conditions, varying factors such as distance from the media or treatment time. The LTP manufacturing protocol was established by detecting ozone or ultraviolet A or B (UV-A, UV-B) derived from LTP. Finally, LTP was prepared with 15 ml of culture media. NTP was treated for 15 min in serum-free culture media distant from the media (about 1~2 cm). The ROS concentration was detected using the CHEMetrics^®^ Kit (Midland, VA, USA) in manufactured LTP. Briefly, LTP was prepared with five samples per each ROS-detection assay. Each sample was prepared with NTP-treated using PBS (15 min/15 ml), and prepared LTP samples were combined into one sample, after which hydrogen, oxygen, and nitrate concentrations were determined according to the manufacturer's instructions. The ozone concentration was measured using a Colorimeter. After measuring pH, LTP was applied to HNC cells in the absence of serum.

### Cell viability assay

SCC-QLL1 and SCC15 cells were seeded at a density of 150 cells/mm^2^. On the following day, cells were washed with PBS and exposed to NTP for 24 hours in each cell culture medium in the absence of serum. Cell viability was checked with a 3-(4,5-dimethylthiazol-2-yl)-2,5-diphenyltetrazolium bromide (MTT) assay kit (Roche, Indianapolis, IN, USA) according to the manufacturer's instructions.

### Western blot analysis

HNC cells were lysed with a radio-immunoprecipitation assay (RIPA) buffer containing phosphate and a protease inhibitor cocktail on ice for 30 min. Following centrifugation at 14,000 × *g* for 20 min at 4°C, proteins in supernatants were separated on a 10% SDS-PAGE gel and transferred to a polyvinylidene difluoride (*PVDF*) membrane. Membranes were blocked with 5% skim milk for 1 hour at room temperature and incubated overnight with primary antibody (1: 1,000) at 4°C. After washing with 0.1% Tween-20 in Tris-buffered saline (TBS-T), membranes were incubated with HRP-conjugated secondary antibody (1: 5,000). Proteins were visualized using ECL reagents (Amersham, Piscataway, NJ, USA) and detected with LAS-4000 (Fuji, Japan). Image densities were quantified with software (http://GelQunat.Net, http://biochemlabsolutions.com/GelQuantNET.html).

### Colony-forming assay

SCC-QLL1 or SCC15 cells (1 × 10^3^ cells) were seeded on six-well plates. LTP or control media containing 10% serum was replaced every other day for 2 weeks. The colony was fixed with cold methanol for 10 min at room temperature and stained with crystal violet. Colony size was measured using the Image J program (http://rsb.info.nih.gov/ij/), as described previously [27].

### Detection of AKT ubiquitination

Ubiquitinated AKT was detected as described previously [14, 26]. Briefly, SCC15 transfected with AKT and Ubi-HA plasmids were washed with PBS, lysed in 200 μl denaturing lysis buffer (50 mM Tris-HCl, pH 7.4, 0.5% SDS, and 70 mM β-mercaptoethanol) by vortexing, and boiled for 15 min at 95°C. The lysates were then diluted with 800 μl buffer A (50 mM NaH_2_PO_4_, 300 mM NaCl, 10 mM imidazole, pH 8.0, protease inhibitor cocktail, and 10 μM MG132) and incubated overnight with 50 μl Ni-NTA beads (Qiagen) at 4°C. Beads were washed five times with buffer B (50 mM NaH_2_PO_4_, 300 mM NaCl, and 20 mM imidazole, pH 8.0), and bound proteins were eluted by boiling in a mixture of 5X SDS-PAGE gel loading buffer and buffer C (50 mM NaH_2_PO_4_, 300 mM NaCl, 250 mM imidazole, pH 8.0) (1:4). Thereafter, exogenously introduced and ubiquitinated AKT were identified with anti-Myc, anti-HA, anti-K48, and anti-K63 antibodies on a Western blot.

### RT-PCR

MUL1, TTC3, and 18s gene expression was estimated using RT-PCR. The total RNA in SCC15 was isolated by TRIzol reagent (Invitrogen). cDNAs were synthesized with 5 μg of total RNAs and ReverTra Ace^®^ qPCR RT Master Mix (TOYOBO Co. Ltd, Japan) according to the manufacturer's instructions. PCR primer sequences were as follows: human *MUL1*, 5′-CAC AAG ATG GTG TGG AAT CG-3′ and 5′-TCA GCA TCT CCT CGG TCT CT-3′; human *TTC3*, 5′-CTT GGC AAT GGA AGA AGC TC-3′ and 5′-AAT GAC CCT TTG GCC AAG TG-3′; human *18s*, 5′-CAC GGA CAG GAT TGA CAG AT-3′ and 5′-CGA ATG GGG TTC AAC GGG TT-3′. PCR products were separated by 1% agarose gel and observed using a LAS4000 (Fuji, Japan).

### RNA interference

MUL1 siRNA was purchased from Ambion (Grand Island, NY, USA). Scrambled RNAs were used as a negative control. A total of 100 pmols of scrambled or MUL1 siRNA were transfected into SCC15 at 70% confluence using RNAiMAX (Invitrogen) according to the manufacturer's instructions. To assess the effect of MUL1 siRNA on AKT ubiquitination, AKT and ubiquitin plasmids were introduced to SCC15 24 hours after siRNA transfection.

### Proximity ligation assay (PLA)

SCC15 or SCC-QLL1 human HNC cells were attached on glass cover slips (Corning, Lowell, MA) at 70% confluence and cultured overnight. On the next day, cells were washed with PBS, treated with NTP, and cultured for an additional 24 hours in the absence of serum. The cells were fixed in 4% paraformaldehyde for 15 min, permeablized with 0.1% Triton X-100, and incubated with 5% BSA for 1 hour at room temperature. Cells were incubated with rabbit anti-MUL1 (1:200) and mouse anti-pan-AKT (1:200) antibodies at 4°C overnight. After washing, the slides were incubated with Duolink PLA Rabbit MINUS and PLA Mouse PLUS proximity probes, and proximity ligation was performed using the Duolink^®^ with PLA^®^ Technology reagent (Sigma, St. Louis, MO) according to the manufacturer's protocol. Fluorescence was detected using an EVOS FL AUTO microscope (Life Technology, NY, USA).

### Measurement of cellular ROS production

To measure cellular ROS production, SCC15 cells were treated with plasma for 24 hours and then treated with 10 μM hydroethidine (HE, Molecular Probes) for 30 min at 37°C. NAC was pretreated for 1 hour before NTP treatment. Fluorescence-stained cells (1 × 10^4^) were quantified using the BD FACSAria III instrument (BD Biosciences).

### TUNEL assay

SCC-QLL1 or SCC15 cells were seeded on cover glass and treated with NTP for 24 hours. DNA fragmentation was analyzed using an *in situ* cell-death detection kit (Roche Molecular Biochemicals, Basel, Switzerland) according to the manufacturer's instructions. Fluorescence was detected using EVOS FL AUTO microscope (Life Technology, NY, USA).

### Immunohistochemistry

Immunohistochemistry was performed on paraffin-embedded tumor sections collected on polylysine-coated slides. Briefly, the specimens were incubated in blocking solution with anti-MUL1 (1:3000) or anti-p-AKT (1:100) antibodies overnight at 4°C. The sections were thoroughly rinsed in PBS and incubated for 2 h at room temperature with SPlink HRP Detection Kit (GBI labs, Muklteo, WA, USA). Immunolabeling was performed after three washes in PBS and stained with Liquid DAB+ Substrate Kit (GBI labs, Mukilteo, WA, USA).

### *In vivo* tumor model

All animal- related experimental procedures and all instances of animal handling were conducted in accordance with the Committee for Ethics in Animal Experiments of the Ajou University School of Medicine. For syngeneic tumor models, 10 female C3H/HeJ mice were purchased from Orient Bio Co. Ltd (SungNam, Korea). Murine SCC7 of HNC cells (1 × 10^6^) resuspended in PBS were administered subcutaneously into the lower right flank after shaving each mouse. After 1 week, when tumors reached ~100 mm in diameter, the mice were randomly divided into two groups (five mice per group) with daily treatment of 200 μl of medium or LTP for 6 days by intratumoral injection. On day 7, the tumors were excised from mice that were killed and used for Western blot assays. For xenograft models, SCC15 (2 × 10^6^) of human HNC cells were inoculated subcutaneously into the lower right flank after shaving each BALB/c nu/nu mice (Orient Bio Co. Ltd, SungNam, Korea) and treated with LTP in the same manner. In xenograft models, LTP was administered 10 times. Tumors were measured using a sliding caliper twice every day, and the volumes (mm^3^) were calculated as described previously [28].

### Statistical analysis

Data are expressed as means ± SD and were analyzed by Student's *t*-test. In some experiments, data were analyzed using ANOVA, followed by the Tukey–Kramer method for multiple comparisons. *P* < 0.05 was considered statistically significant.

## SUPPLEMENTARY FIGURES AND TABLES


