# Supplementary material for: Non-thermal plasma induces AKT degradation through turn-on the MUL1 E3 ligase in head and neck cancer
Source: Oncotarget. 2015 Sep 30;6(32):33382–96. doi: 10.18632/oncotarget.5407 (PMC4741773; doi:10.18632/oncotarget.5407)
Supplement: Supplementary file 1 [file oncotarget-06-33382-s001.pdf]

SUPPLEMENTARY FIGURES AND TABLES

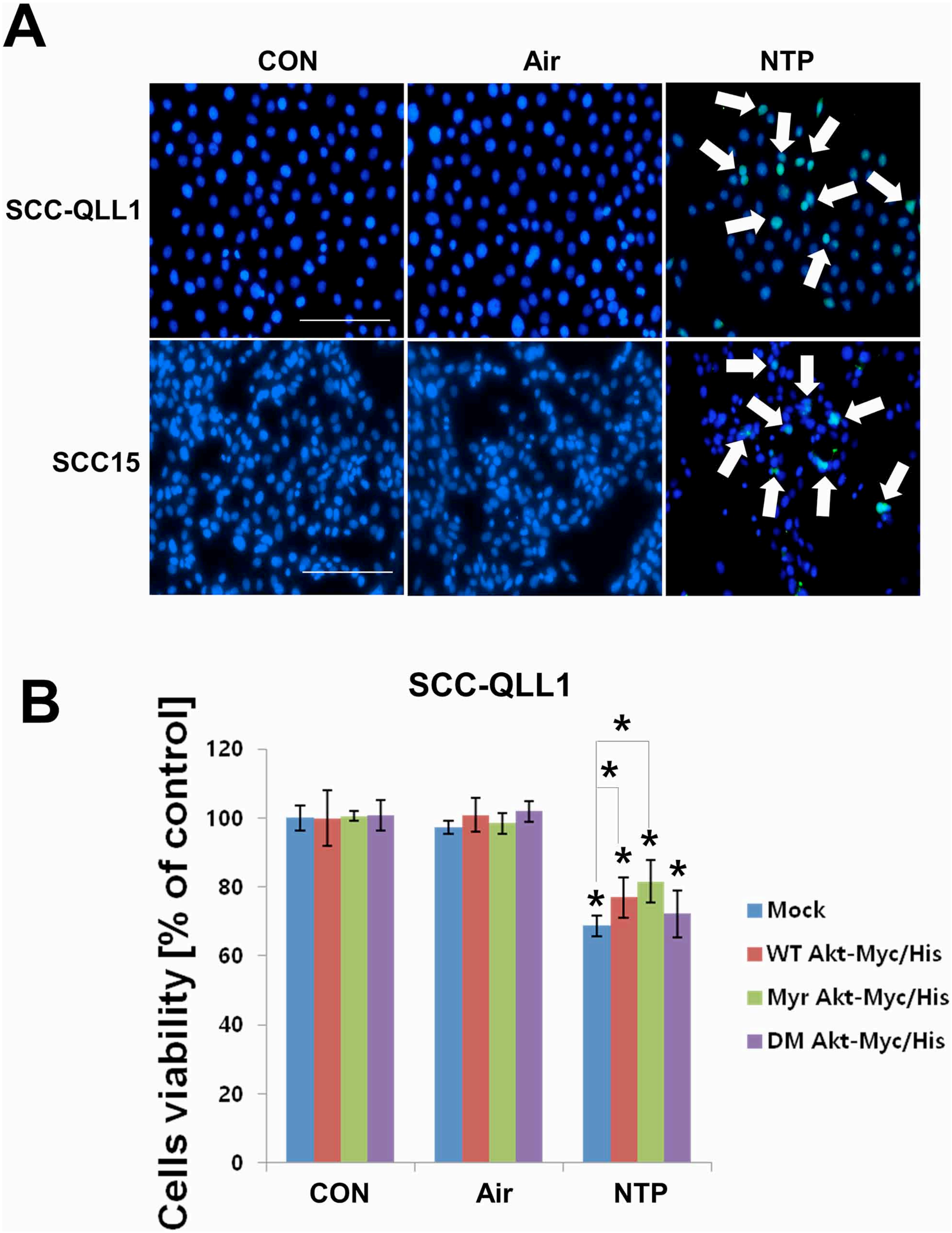

Supplementary Figure S1: NTP induced HNC apoptotic cell death and active AKT prevented NTP-induced cytotoxicity. A. SCC-QLL1 cells were seeded at cover slip and then, NTP was treated for 24 hours. Cell death was performed with TUNEL staining. Arrows indicates TUNEL positive cells (green). Scale bar = 50 μm. **B.** Each indicated plasmids were transfected into SCC-QLL1 cells and then, NTP was treated for 24 hours. Cells viability was determined with MTT assay. Asterisks indicate statistically significant differences ($P < 0.05$). Data are expressed as means ± SD.

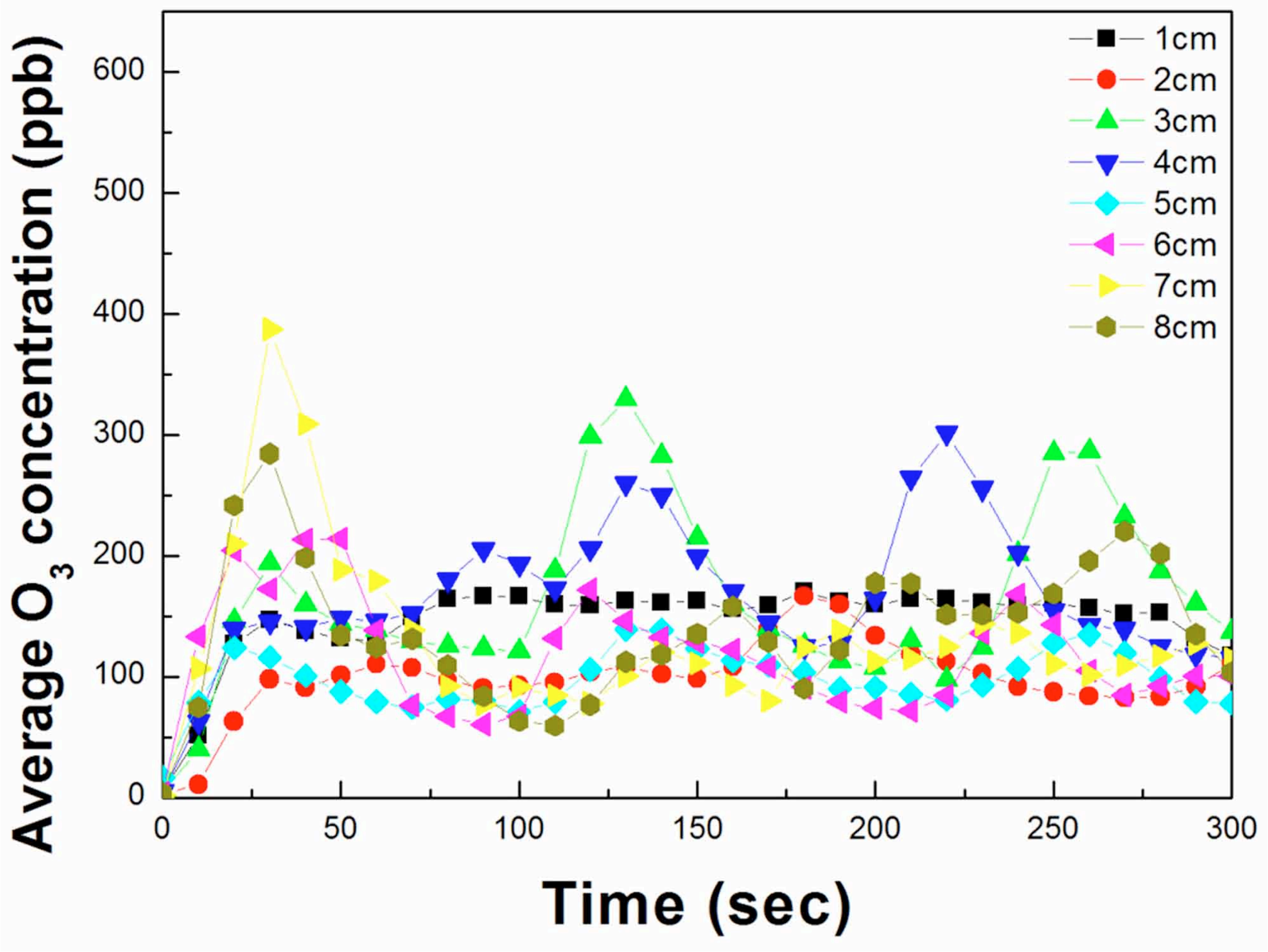

Supplementary Figure S2: The detection of ozone concentration for LTP preparation optimize. The method used to prepare liquid-type NTP (LTP) was optimized by testing under several different conditions, varying factors such as distance from the media or treatment time. The ozone concentration was measured by Colorimeter.

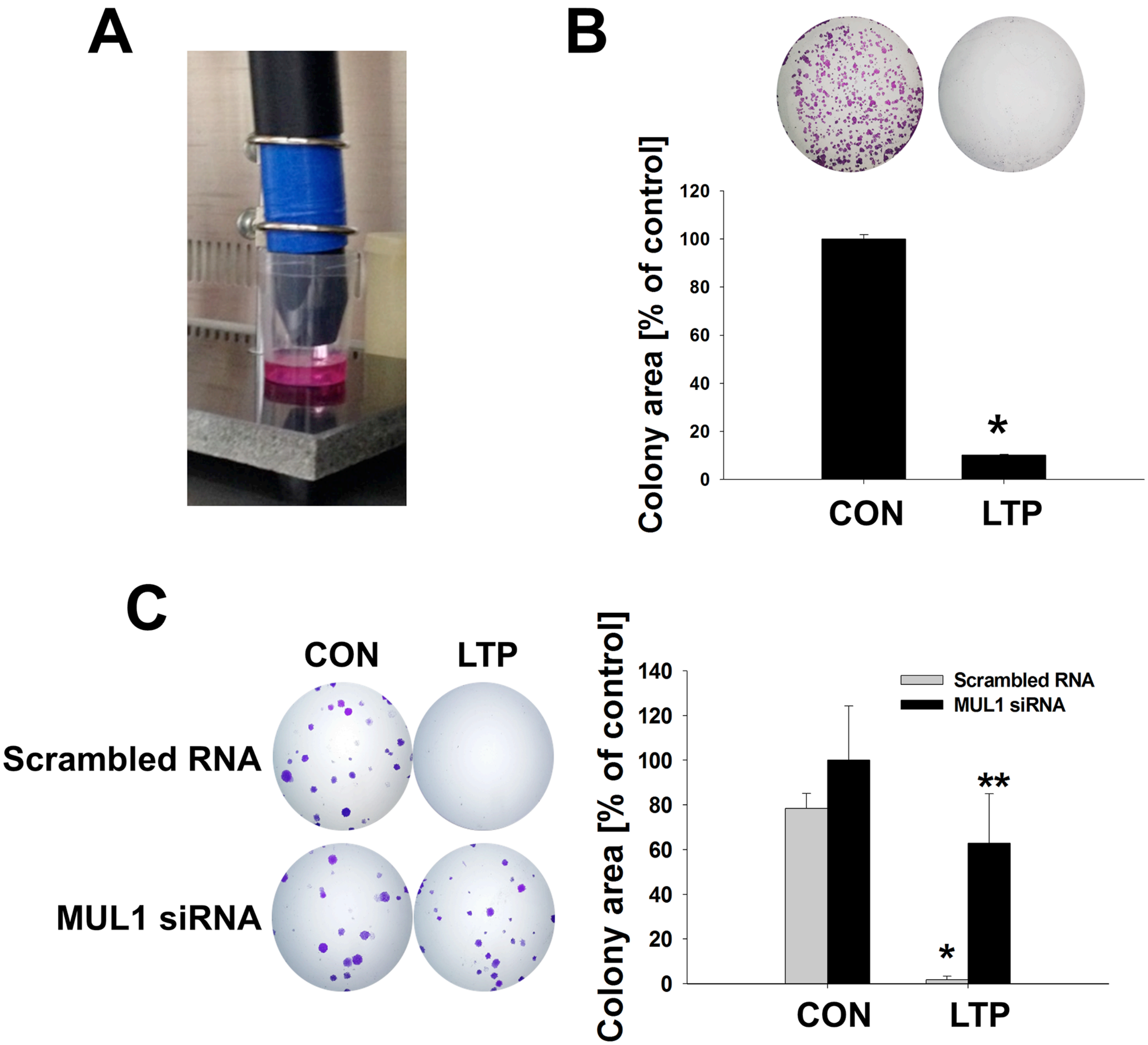

Supplementary Figure S3: Liquid type of plasma (LTP) preparation and evaluation of anti-cancer effect. A. Scheme of LTP preparation. NTP (He + O^2, 4 kV) was treated for 15 min at 15 ml of media from the 1~2 cm distance in the absence of serum. **B.** 1,000 cells of SCC-QLL1 seeded on 6-well plate and LTP contained 10% serum was replaced every other day. Colony forming growth was stained with crystal violet. Asterisks indicate statistically significant differences ($P < 0.05$). Data are expressed as means ± SD. **C.** LTP -induced inhibition of colony forming growth was prevented in MUL1 knock-downed cells. Scrambeld RNA or MUL1 trasnfected SCC15 cells were seeded at 6-well plate at a density of 1,000 cells. Control media or LTP contained with 10% of serum was replaced at every other day. Asterisks indicate statistically significant differences ($P < 0.05$) between LTP -untreated and LTP treated groups (* vs CON; ** vs scrambled RNA). Data are expressed as means ± SD.

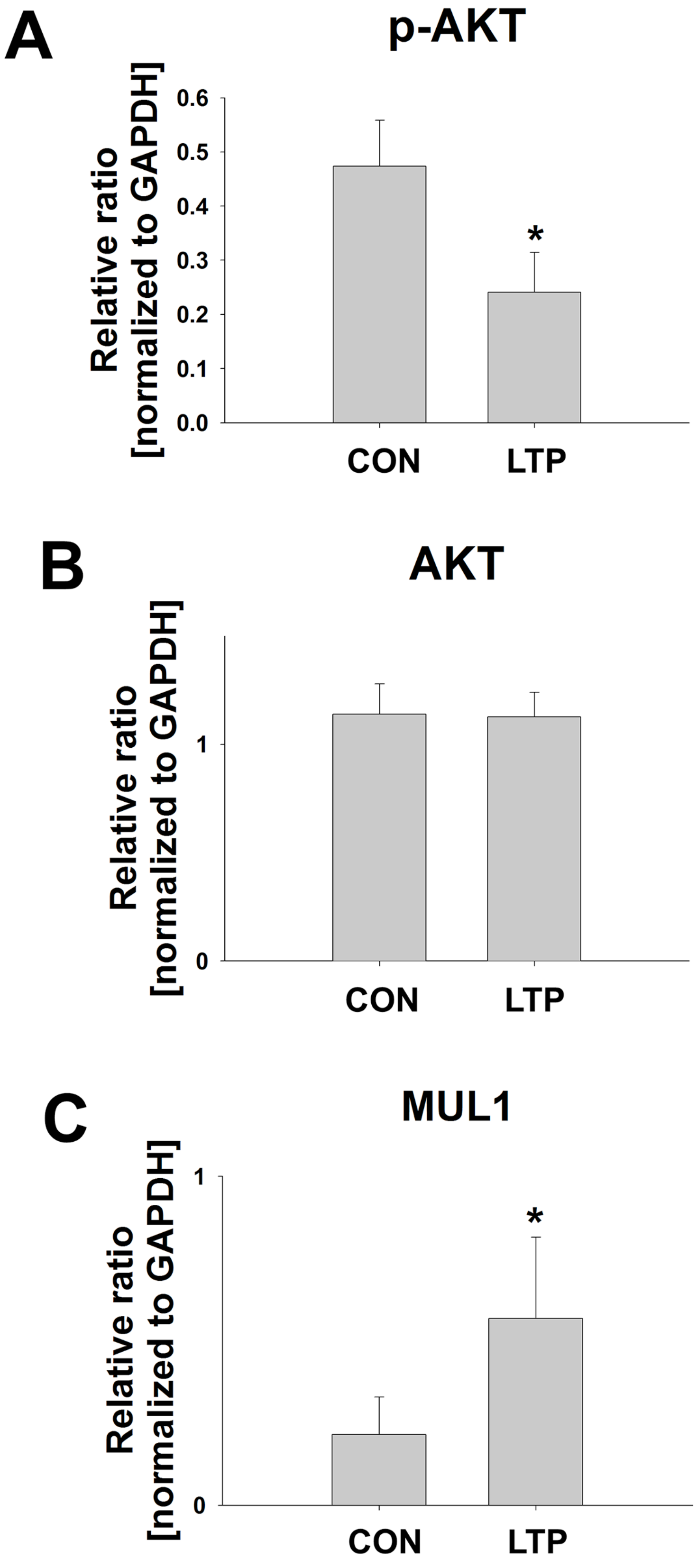

Supplementary Figure S4: p-AKT, AKT or MUL1 quantitation results. In Figure 6D western blot analysis results were quantified. **A.** p-AKT. **B.** AKT. **C.** MUL1. Asterisks indicate statistically significant differences ($P < 0.05$, $n = 8$) between LTP -untreated and LTP treated groups. Data are expressed as means ± SD.

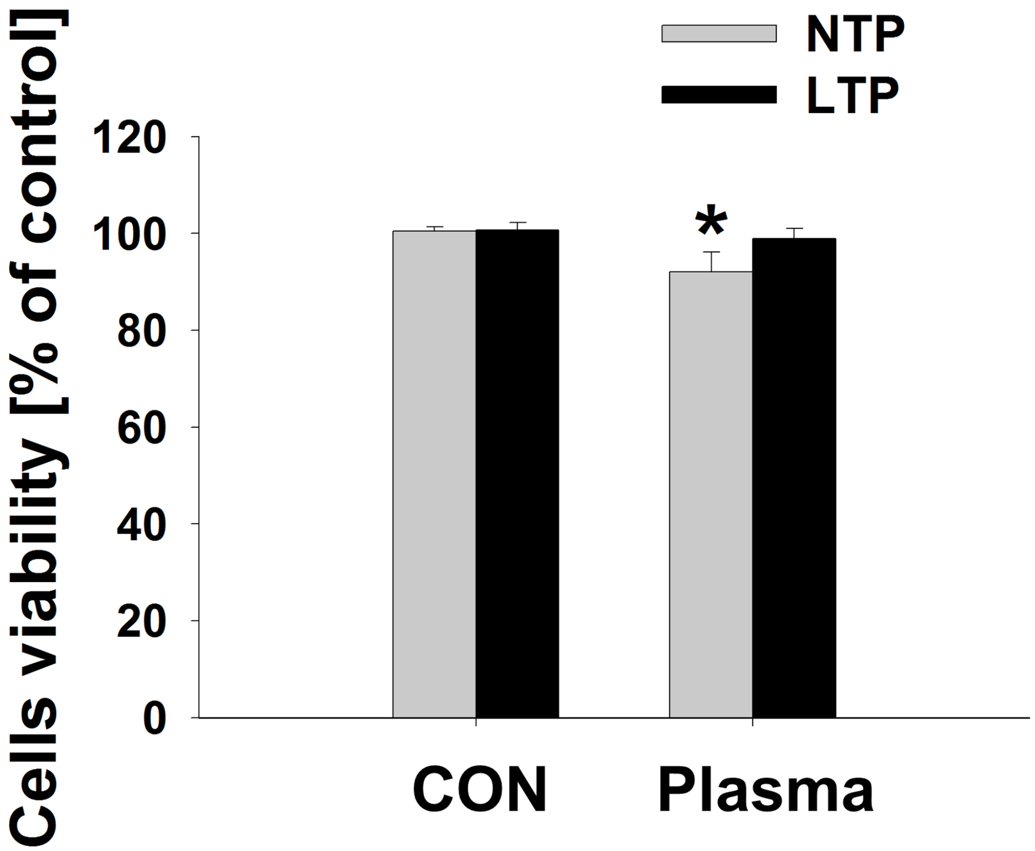

Supplementary Figure S5: Evaluation of plasma effect in human normal lung fibroblasts (HNLFs). HNLFs were seed at 48-well plate and treated with NTP or LTP for 24 hours. Cells viability were determined by MTT assay. Asterisks indicate statistically significant differences ($P < 0.05$) between NTP or LTP -untreated and treated groups. Data are expressed as means ± SD.

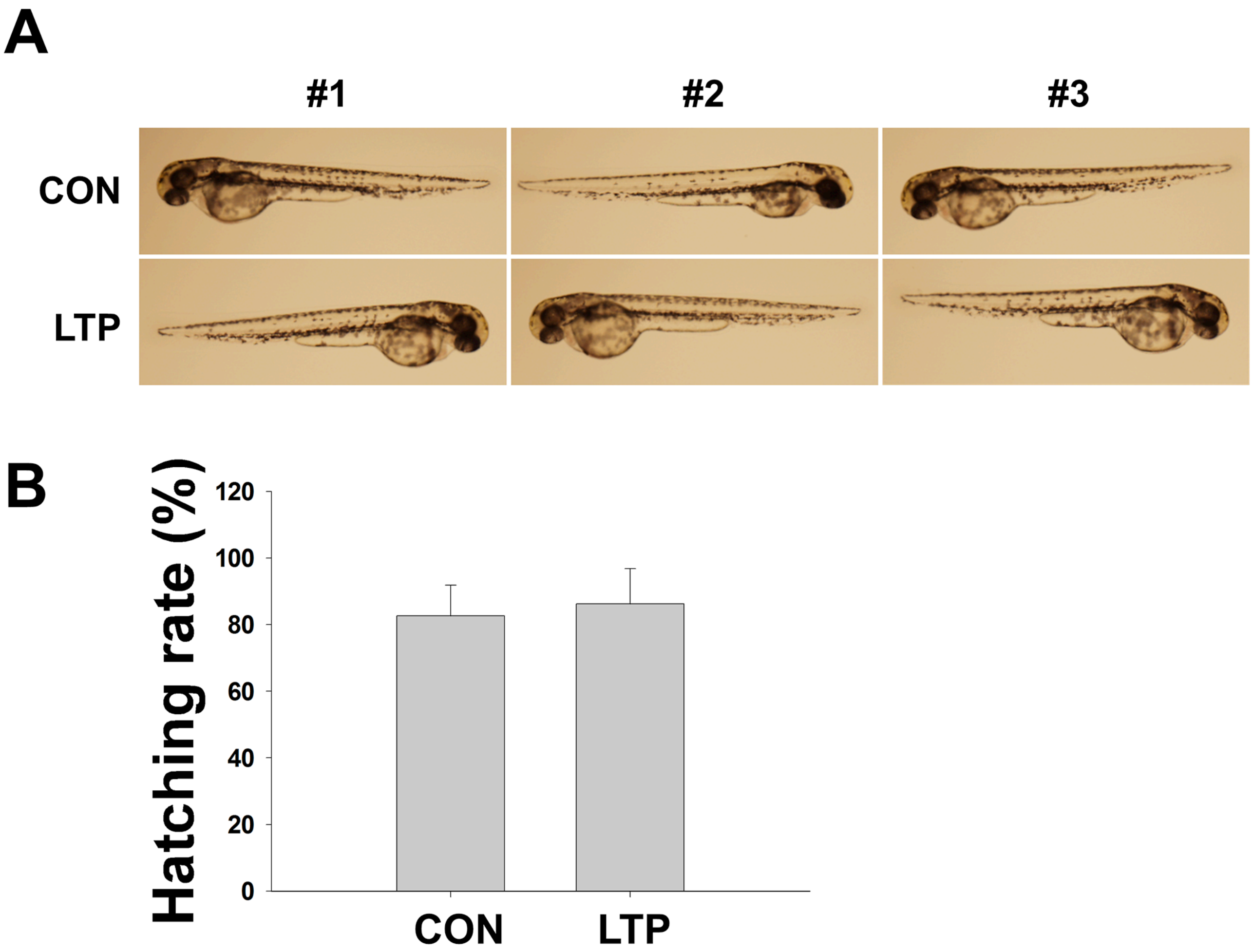

Supplementary Figure S6: Fish embryo toxicity test (FET) of LTP. Fresh zebrafish eggs (30 eggs) were incubated in LTP-treated egg water at 37°C for 3 days. On 3 day, fishes were pictured randomly 5 fields each group **A.** and quantified eggs hatching rate (**B.** n = 3). Data are expressed as means ± SD.

Supplementary Table S1: Detection of UV A or UV B for NTP preparation optimize

Distance	UV A (315 ~ 400 nm)	UV B (280 ~ 315 nm)
1 cm	337 mW / cm^2	156 mW / cm^2
2 cm	144 mW / cm^2	49 mW / cm^2
3 cm	98 mW / cm^2	31 mW / cm^2
4 cm	80 mW / cm^2	19 mW / cm^2
5 cm	47 mW / cm^2	15 mW / cm^2
6 cm	40 mW / cm^2	12 mW / cm^2
7 cm	30 mW / cm^2	9 mW / cm^2
8 cm	27 mW / cm^2	7 mW / cm^2

Supplementary Table S2: Detection of ozone or ROS in LTP

ROS	Concentration (ppm ± SD)
Ozone (O_3)	1.154 ± 0.00145
Hydrogen (H_2O_2)	1.8333 ± 0.3152
Oxygen (O_2)	4.767 ± 0.1453
Nitrate (No)	0.1673 ± 0.1663

Data are expressed as means ± SD.
